# Diet, Obesity and Cardiovascular Health: Molecular Mechanisms, Clinical Evidence and Future Perspectives

**DOI:** 10.3390/jcm15197428

**Published:** 2026-09-24

**Authors:** Francesca Marino, Maria Teresa Savo, Dan Alexandru Cozac, Domenico Galzerano, Mohammed El Sabah, Gabriele Cordoni, Donatella Tansella, Maria Martina Palmisano, Naji Kholaif, Dania Mohty, Giovanni Di Salvo, Hani Al Sergani, Valeria Pergola

**Affiliations:** 1 Department of Educational, Psychological and Communication Sciences, University Suor Orsola Benincasa, 80135 Naples, Italy; 2 UNESCO Chair “Education for Health and Sustainable Development”, University of Naples Federico II, 80138 Naples, Italy; 3 PhD Program in Translation Specialistic Medicine “G.B. Morgagni”, Curriculum “Cardiovascular Sciences”, University of Padua, 35128 Padua, Italy; 4 Physiology Department, George Emil Palade University of Medicine, Pharmacy, Science, and Technology of Targu Mures, 540142 Targu Mures, Romania; 5 Heart Centre of Excellence, King Faisal Specialist Hospital & Research Center, 12713 Riyadh, Saudi Arabia; 6 College of Medicine, Alfaisal University, 11533 Riyadh, Saudi Arabia; 7 Department of Heart, Lungs and Vessels, Montpellier University Hospital, CHU Arnaud de Villeneuve, 34000 Montpellier, France; 8 Cardiology Unit, Mater Dei Hospital, 70125 Bari, Italy; 9 Department of Cardio-Thoracic Sciences and Public Health, University of Padua, 35128 Padua, Italy; 10 Paedriatic Cardiology Unit, Department of Woman’s and Child’s Health, University Hospital of Padua, 35128 Padua, Italy

**Keywords:** obesity, cardiovascular disease, Mediterranean diet, dietary patterns, mitochondrial dysfunction, inflammation, endothelial dysfunction, gut microbiota, precision nutrition, cardiometabolic health

## Abstract

**Background:** Obesity and unhealthy dietary habits are among the leading modifiable determinants of cardiovascular disease, acting through biological mechanisms that extend far beyond excess body weight. Adipose tissue dysfunction, chronic low-grade inflammation, oxidative stress, endothelial impairment, mitochondrial dysfunction, and gut microbiota dysbiosis contribute to the development and progression of cardiometabolic disease. Consequently, nutrition should be regarded as a key biological modulator of cardiovascular risk, capable of influencing multiple interconnected metabolic and molecular pathways. **Methods:** This narrative review provides a critical and translational overview of the current evidence linking diet, obesity, and cardiovascular disease. We critically evaluated epidemiological studies, landmark randomized controlled trials, prospective cohort studies, meta-analyses, international guidelines, and relevant mechanistic investigations to examine the impact of major dietary patterns, obesity-related immunometabolic alterations, and emerging precision nutrition strategies on cardiovascular prevention and management. **Results:** Among currently available dietary models, the Mediterranean diet has one of the most extensive randomized evidence bases for cardiovascular prevention, including trials demonstrating reductions in major adverse cardiovascular events and other clinically relevant cardiovascular outcomes. Other healthy dietary patterns, including the Dietary Approaches to Stop Hypertension (DASH), healthy plant-based, and Nordic diets, consistently improve intermediate cardiometabolic outcomes, although evidence for hard cardiovascular endpoints is less robust. Conversely, Western dietary patterns characterized by high consumption of ultra-processed foods are associated with visceral adiposity, insulin resistance, vascular inflammation, mitochondrial dysfunction, endothelial injury, and increased cardiovascular risk. **Conclusions:** Contemporary evidence supports nutrition as a cornerstone of cardiovascular prevention and obesity management, with benefits may extend beyond weight reduction through modulation of key biological pathways involved in cardiometabolic disease.

## 1. Introduction

Cardiovascular disease (CVD) remains the leading cause of death worldwide, accounting for nearly one-third of all global mortality and imposing an enormous clinical, social, and economic burden on healthcare systems. Despite remarkable advances in pharmacological therapies and interventional cardiology that have substantially improved survival after acute cardiovascular events, the global epidemic of obesity and metabolic disorders continues to threaten these achievements, limiting progress in cardiovascular prevention and increasing healthcare costs worldwide [1,2]. Indeed, high body mass index (BMI) remains one of the fastest-growing contributors to the global burden of disease, with age-standardized disability-adjusted life-year (DALY) rates attributable to elevated BMI increasing by 15.7% between 2000 and 2021 [2].

Obesity is now recognized as a chronic, relapsing, multifactorial disease rather than simply a consequence of excessive body weight. Recent consensus criteria distinguish clinical obesity, a chronic, systemic illness characterized by functional alterations in tissues and organs due to excess adiposity, from preclinical obesity, in which excess adiposity is present with preserved organ function but a generally increased risk of subsequent disease [3]. Beyond adipose tissue expansion, obesity is characterized by profound metabolic, endocrine, inflammatory, and immunological alterations that promote cardiovascular injury. Dysfunctional adipose tissue acts as an active endocrine organ, releasing pro-inflammatory cytokines, chemokines, and adipokines that contribute to insulin resistance, endothelial dysfunction, oxidative stress, vascular remodeling, and accelerated atherosclerosis [4].

Among the biological processes linking obesity to CVD, mitochondrial dysfunction has emerged as a central pathogenic mechanism. Altered mitochondrial dynamics, impaired oxidative phosphorylation, excessive reactive oxygen species (ROS) production, defective mitophagy, and reduced metabolic flexibility contribute to chronic low-grade inflammation and progressive cellular dysfunction. Importantly, these alterations affect not only adipose tissue, skeletal muscle, and the liver but also the cardiovascular system and the central nervous system, perpetuating a vicious cycle of obesity, metabolic dysregulation, and cardiovascular risk [4].

Alongside these advances in pathophysiological understanding, nutrition has shifted from being viewed primarily as a determinant of caloric balance to being recognized as a powerful regulator of cellular metabolism and cardiovascular biology. Diet and obesity should not be regarded as independent cardiovascular risk factors, as dietary patterns are among the principal determinants of both the development and the metabolic consequences of excess adiposity. Chronic consumption of energy-dense, nutrient-poor foods promotes positive energy balance, visceral fat accumulation, and adipose-tissue dysfunction, thereby amplifying insulin resistance, systemic inflammation, oxidative stress, and endothelial injury. However, body weight does not exclusively mediate this relationship. Dietary components influence multiple biological pathways involved in cardiovascular homeostasis, including inflammatory signaling, endothelial nitric oxide bioavailability, mitochondrial bioenergetics, immune-cell activation, gut microbiota composition, oxidative stress, adipose tissue expansion, and epigenetic regulation of cardiometabolic genes. Consequently, the cardiovascular effects of nutrition extend well beyond weight reduction, supporting the concept that dietary interventions may directly modulate disease mechanisms and improve cardiovascular outcomes independently of body weight changes [5].

Among currently available dietary models, the Mediterranean diet is among the most extensively investigated patterns for cardiovascular prevention and is supported by substantial evidence from randomized controlled trials and prospective cohort studies [6,7,8]. Other dietary patterns, including the Dietary Approaches to Stop Hypertension (DASH), healthy plant-based, Nordic, and Mediterranean–DASH Intervention for Neurodegenerative Delay (MIND) diets, have also demonstrated favorable effects on blood pressure, lipid metabolism, glycemic control, endothelial function, and systemic inflammation, although the level of evidence for hard cardiovascular outcomes is generally less robust [9,10,11]. Despite differences in food composition and cultural background, these dietary models share fundamental biological principles, including a high intake of minimally processed plant-derived foods, unsaturated fats, dietary fiber, and bioactive compounds, together with limited consumption of ultra-processed foods (UPFs) and refined carbohydrates.

This narrative review aims to provide a comprehensive and translational overview of the current evidence linking dietary habits, obesity, and CVD. Rather than considering diet, obesity, and cardiovascular disease as separate or exclusively pairwise relationships, this review examines them as components of an interconnected cardiometabolic continuum. In this framework, dietary exposures may influence cardiovascular risk indirectly by promoting or reversing adipose-tissue dysfunction, and directly through effects on inflammation, oxidative stress, endothelial function, mitochondrial homeostasis, and the gut microbiota, partly independent of changes in body weight. Particular attention is therefore given to distinguishing weight-dependent from weight-independent cardiovascular effects of nutrition and to identifying areas in which mechanistic evidence has not yet translated into cardiovascular outcome data. Particular emphasis is placed on the molecular mechanisms through which nutrition influences cardiometabolic health—including chronic inflammation, endothelial dysfunction, mitochondrial impairment, and gut microbiota alterations—as well as on the clinical evidence supporting major dietary patterns for cardiovascular prevention. Finally, we discuss emerging concepts in precision nutrition and personalized cardiovascular medicine as potential future directions for improving long-term cardiometabolic health and reducing the global burden of cardiovascular disease. To support this narrative review, PubMed/MEDLINE, Scopus, and Web of Science were searched up to July 2026 using combinations of terms related to diet and dietary patterns, obesity and adiposity, cardiovascular disease, inflammation, oxidative stress, endothelial dysfunction, mitochondrial dysfunction, gut microbiota, and precision nutrition. We prioritized randomized controlled trials, prospective cohort studies, systematic reviews and meta-analyses, international guidelines and consensus documents, and mechanistic studies directly relevant to the scope of the review. Studies were selected based on their relevance to the main thematic areas addressed in the manuscript, with preference given to higher-level clinical evidence where available and to mechanistic or preclinical studies when needed to support biological interpretation. Because this was a narrative rather than a systematic review, we did not perform a formal study-selection protocol, risk-of-bias assessment, or quantitative evidence synthesis.

## 2. Epidemiological Links Between Diet, Obesity and Cardiovascular Disease

The worldwide prevalence of obesity has nearly tripled over the last five decades, becoming one of the most significant contributors to the global burden of CVD. According to the World Health Organization, more than one billion individuals are currently living with obesity, while overweight and obesity collectively account for millions of deaths annually through their association with CVD, type 2 diabetes mellitus, chronic kidney disease, and several malignancies [12]. Severe obesity is associated with a significant reduction in life expectancy of 9.1 years in men and 7.7 years in women [13]. The increasing availability of calorie-dense, nutrient-poor foods, combined with reduced physical activity and profound lifestyle changes, has accelerated the development of cardiometabolic disorders across all age groups [3].

Visceral adiposity has emerged as the strongest anthropometric predictor of cardiovascular morbidity and mortality. Compared to subcutaneous fat, visceral adipose tissue exhibits greater lipolytic activity, enhanced secretion of pro-inflammatory cytokines—including tumor necrosis factor-α (TNF-α), interleukin-6 (IL-6), and monocyte chemoattractant protein-1 (MCP-1)—and reduced production of protective adipokines such as adiponectin. These alterations promote endothelial dysfunction, insulin resistance, vascular inflammation, and accelerated atherogenesis, independently of BMI [14]. Within this framework, epidemiological data offer initial support for a partially mediated model: excess adiposity, particularly visceral adiposity, appears to account for part, but not all of the association between adverse dietary patterns and cardiovascular disease, since diet-quality associations with cardiovascular risk frequently persist after adjustment for BMI and other conventional risk factors, suggesting additional weight-independent mechanisms. However, persistence after multivariable adjustment does not establish an adiposity-independent causal effect, because residual confounding, dietary-measurement error, reverse causation, and unmeasured socioeconomic or behavioral factors may remain.

Large prospective cohort studies have consistently demonstrated a dose-dependent relationship between obesity and cardiovascular events. The Prospective Urban Rural Epidemiology study, involving participants from diverse socioeconomic settings across multiple continents, confirmed that poor diet quality and central obesity significantly increase cardiovascular risk regardless of geographic region or national income level [15]. Similarly, the European Prospective Investigation into Cancer and Nutrition cohort highlighted the protective role of high consumption of fruits, vegetables, legumes, whole grains, and olive oil, whereas dietary patterns characterized by processed meat, refined carbohydrates, and saturated fats were associated with increased cardiovascular mortality [16].

Recent nutritional epidemiology indicates that two individuals with similar BMI may exhibit markedly different cardiovascular risk profiles depending on dietary composition. Diets rich in minimally processed plant-derived foods are consistently associated with lower circulating inflammatory biomarkers, improved lipid profiles, enhanced endothelial function, and reduced incidence of coronary artery disease, even after adjustment for body weight and traditional cardiovascular risk factors [17]. Conversely, Western dietary patterns rich in UPFs, sugar-sweetened beverages, trans fats, and excessive sodium intake are associated with chronic inflammation, metabolic dysfunction, hypertension, dyslipidemia, and higher cardiovascular risk [18].

The relationship between obesity and CVD is already evident during childhood. Pediatric obesity has become a major public health concern because cardiovascular remodeling begins early in life and frequently persists into adulthood [19]. Excess adiposity during childhood is associated with increased arterial stiffness, endothelial dysfunction, left ventricular hypertrophy, impaired vascular compliance, and early metabolic abnormalities that predispose individuals to premature CVD [20]. Consequently, preventive nutritional interventions should ideally begin early in life rather than after overt cardiometabolic disease develops. Children with congenital heart disease represent an especially vulnerable population, in whom obesity further aggravates hemodynamic burden and negatively affects long-term prognosis [20].

Population-based and interventional studies indicate that a 5–10% reduction in body weight is associated with clinically meaningful improvements in blood pressure, triglyceride concentrations, insulin sensitivity, hepatic steatosis, and inflammatory markers [21,22]. Greater weight loss generally produces additional cardiometabolic benefits; however, evidence supporting a specific weight-loss threshold above 15% for reducing major adverse cardiovascular events MACE remains limited [23]. In patients with overweight or obesity and established CVD, cardiovascular outcome trials have nevertheless demonstrated that selected weight-management interventions can reduce the incidence of MACE [24].

## 3. Molecular Mechanisms Linking Diet, Obesity and Cardiovascular Health

### 3.1. Adipose Tissue Dysfunction: From Energy Storage to Endocrine Organ

The partial and incomplete mediation observed at the population level has a plausible biological basis. Unhealthy dietary exposures promote adipocyte hypertrophy and adipose-tissue dysfunction, which activate inflammatory, oxidative, metabolic, and endothelial pathways that favor cardiovascular injury. Several dietary components, however, also act on these same pathways directly, largely independently of measurable changes in adiposity.

Adipose tissue is now recognized as a highly active endocrine and immunometabolic organ rather than a passive energy reservoir. Under physiological conditions, adipocytes regulate systemic energy balance by secreting adipokines, cytokines, growth factors, and extracellular vesicles that coordinate glucose homeostasis, lipid metabolism, appetite regulation, vascular function, and immune responses [25]. As obesity progresses, adipocyte hypertrophy exceeds the capacity of adipose tissue remodeling, leading to local hypoxia, extracellular matrix expansion, fibrosis, and progressive infiltration of immune cells. Macrophages shift from an anti-inflammatory M2 phenotype toward a pro-inflammatory M1 phenotype, accompanied by increased recruitment of T lymphocytes, neutrophils, dendritic cells, and mast cells. This cellular remodeling transforms adipose tissue into a chronic source of inflammatory mediators, including TNF-α, interleukin-1β (IL-1β), IL-6, MCP-1, resistin, and leptin [26] (Figure 1).

Conversely, circulating concentrations of adiponectin—one of the principal anti-inflammatory and vasculoprotective adipokines—decline markedly with increasing adiposity. Reduced adiponectin impairs AMP-activated protein kinase signaling, decreases endothelial nitric oxide synthase (eNOS) activity, promotes insulin resistance, and facilitates vascular inflammation, thereby accelerating atherosclerotic plaque formation [27].

### 3.2. Chronic Inflammation and Immunometabolic Crosstalk

Unlike the acute inflammatory response observed during infection or tissue injury, obesity is characterized by persistent activation of innate and adaptive immune pathways that remain chronically stimulated for years. Hypertrophic adipocytes release danger-associated molecular patterns (DAMPs), free fatty acids, ceramides, and oxidized lipids that activate Toll-like receptor 4 (TLR4) and nucleotide-binding oligomerization domain-like receptor protein 3 (NLRP3) inflammasomes in macrophages and endothelial cells. This signaling cascade activates nuclear factor kappa B (NF-κB), enhances transcription of pro-inflammatory cytokines, and amplifies oxidative stress [28]. The CANTOS trial convincingly demonstrated the clinical relevance of inflammation, as selective inhibition of IL-1β significantly reduced recurrent MACE independently of lipid lowering, confirming inflammation as a therapeutic target in atherosclerotic disease [29].

### 3.3. Oxidative Stress and Endothelial Dysfunction

Oxidative stress represents another critical interface between obesity and CVD. Under physiological conditions, ROS participate in intracellular signaling and vascular homeostasis. However, excessive ROS production overwhelms endogenous antioxidant defenses, leading to lipid peroxidation, protein oxidation, DNA damage, and impaired cellular function [30]. Multiple enzymatic systems contribute to ROS generation in obesity, including NADPH oxidases, xanthine oxidase, uncoupled eNOS, and dysfunctional mitochondria. Elevated circulating free fatty acids further stimulate oxidative stress through enhanced β-oxidation and electron transport chain overload [30]. The vascular endothelium is particularly vulnerable to oxidative injury. ROS rapidly inactivate nitric oxide, reducing its bioavailability and impairing endothelium-dependent vasodilation. Endothelial dysfunction subsequently promotes leukocyte adhesion, platelet activation, vascular permeability, and smooth muscle cell migration, all of which contribute to early atherogenesis [31] (Figure 2).

### 3.4. Mitochondrial Dysfunction: A Central Hub Connecting Obesity and Cardiovascular Disease

Obesity is associated with reduced mitochondrial biogenesis, impaired oxidative phosphorylation, defective fatty acid oxidation, increased mitochondrial fragmentation, altered fusion–fission dynamics, and progressive accumulation of damaged mitochondria due to defective mitophagy. Collectively, these alterations reduce metabolic flexibility while increasing ROS generation, ultimately promoting chronic inflammation and insulin resistance [32].

Experimental studies further demonstrate that mitochondrial dysfunction precedes overt metabolic disease, suggesting that impaired mitochondrial quality control may represent an initiating event rather than merely a consequence of obesity. Dysregulation of key metabolic regulators—including peroxisome proliferator-activated receptor gamma coactivator-1α (PGC-1α), sirtuin-1 (SIRT1), AMP-activated protein kinase, and mitochondrial transcription factor A—reduces oxidative capacity and accelerates cardiometabolic deterioration [33].

Importantly, several dietary interventions directly improve mitochondrial function [34]. Caloric restriction, Mediterranean dietary patterns, intermittent fasting, omega-3 fatty acids, dietary polyphenols, and regular physical activity all stimulate mitochondrial biogenesis by activating AMPK and SIRT1 signaling pathways while reducing oxidative stress and enhancing mitophagy. These observations suggest that preserving mitochondrial quality may be a principal mechanism by which healthy dietary habits reduce cardiovascular risk [4].

### 3.5. Gut Microbiota and the Gut–Heart Axis

The gut–heart axis refers to the bidirectional interaction between the gastrointestinal tract, its resident microbiota, and the cardiovascular system [35]. Gut microorganisms may influence cardiovascular function by producing bioactive metabolites, including short-chain fatty acids (SCFAs), trimethylamine N-oxide (TMAO), and secondary bile acids, which can enter systemic circulation and exert metabolic, inflammatory, and direct cardiovascular effects [36].

Fermentable dietary fibers are metabolized by the gut microbiota into SCFAs, particularly acetate and butyrate, which support intestinal barrier integrity and may exert anti-inflammatory and cardiovascular-protective effects [37]. Experimental studies suggest that SCFAs reduce blood pressure, cardiac hypertrophy, and fibrosis by activating host receptors [38]. Small human trials have generally shown reductions in blood pressure after colonic SCFA delivery or supplementation with fermentable fiber, although findings are not entirely consistent and may depend on the site and route of SCFA absorption [39].

Elevated circulating TMAO concentrations have been associated with increased platelet reactivity, endothelial dysfunction, vascular inflammation, adverse outcomes in both acute decompensated and chronic heart failure, as well as with greater severity of diastolic dysfunction [39,40]. Community-based studies have also linked higher concentrations of TMAO and related metabolites, including choline, carnitine, and crotonobetaine, to an increased risk of incident heart failure [40]. Dietary patterns can modulate gut microbial composition and TMAO production, with plant-based diets generally associated with greater microbial diversity and lower circulating TMAO levels. Conversely, red meat intake increases TMAO by providing choline and carnitine substrates, enhancing microbial trimethylamine production, and reducing renal TMAO excretion [41].

Dysbiosis may also impair intestinal barrier integrity, promoting the translocation of microbial products such as lipopolysaccharide, a component of Gram-negative bacteria. Subsequent activation of TLR4–mediated pathways may contribute to systemic inflammation. Studies have observed increased intestinal permeability and circulating inflammatory mediators in patients with heart failure, supporting the potential clinical relevance of gut-derived mechanisms in this condition.

### 3.6. Epigenetic Regulation

Emerging evidence suggests that many dietary effects of the cardiovascular system are mediated by epigenetic mechanisms, including DNA methylation, histone modifications, chromatin remodeling, and regulation by non-coding RNAs. Nutrients and bioactive compounds can influence gene expression without altering DNA sequence, thereby contributing to long-term cardiometabolic adaptation [42]. Polyphenols, omega-3 fatty acids, folate, B vitamins, and several phytochemicals have shown epigenetic activity that can modulate inflammatory pathways, oxidative stress responses, mitochondrial metabolism, and endothelial function.

## 4. Mediterranean Diet: Evidence for Cardiovascular Prevention

Among the various dietary models investigated over the past decades, the Mediterranean diet has emerged as one of the most extensively studied dietary patterns for cardiovascular prevention, supported by both randomized clinical trials and observational evidence. Originally described through the pioneering observations in the Seven Countries Study, the Mediterranean diet reflects the traditional eating habits of populations in Southern Europe during the mid-twentieth century. These populations had remarkably low rates of coronary heart disease despite moderate total fat intake, suggesting that dietary quality, rather than total fat intake, was the main determinant of cardiovascular protection [43].

Contemporary definitions of the Mediterranean dietary pattern emphasize high consumption of vegetables, fruits, legumes, whole grains, nuts, seeds, and extra-virgin olive oil as the primary dietary fat source. Moderate intake of fish, seafood, fermented dairy products, and poultry is encouraged, whereas red and processed meat, refined sugars, highly processed foods, and saturated fats are consumed sparingly. Moderate wine consumption during meals, regular physical activity, adequate sleep, and social eating habits are traditionally considered integral components of the Mediterranean lifestyle rather than isolated nutritional recommendations [44].

### 4.1. Clinical Evidence from Randomized Controlled Trials

The strongest evidence supporting the cardiovascular efficacy of the Mediterranean diet originates from randomized clinical trials, summarized in Table 1. From a clinical perspective, dietary interventions may operate in two distinct settings: primary prevention, aimed at reducing the development of cardiovascular disease in at-risk individuals, and secondary prevention in patients with established disease. Although these contexts overlap mechanistically, their target populations, therapeutic objectives, and clinically relevant outcomes differ and should therefore be considered separately. Evidence from Prevención con Dieta Mediterránea (PREDIMED) supports its efficacy in primary prevention among individuals at high cardiovascular risk, whereas the Lyon Diet Heart Study and CORDIOPREV demonstrate benefits in secondary prevention [7,8,45]. The PREDIMED trial fundamentally changed nutritional cardiology by showing that a Mediterranean dietary intervention supplemented with either extra-virgin olive oil or mixed nuts significantly reduced the incidence of MACE among individuals at high cardiovascular risk [7]. Importantly, these benefits occurred despite only modest changes in body weight, indicating that dietary composition exerts cardioprotective effects independently of weight reduction.

The Lyon Diet Heart Study evaluated secondary cardiovascular prevention in patients with previous myocardial infarction: participants assigned to a Mediterranean dietary intervention experienced a remarkable reduction in recurrent myocardial infarction, cardiovascular mortality, and overall mortality compared to those following a prudential diet suggested by their attending physicians [46].

More recently, the CORDIOPREV trial expanded these findings by comparing Mediterranean and low-fat dietary interventions among patients with established coronary artery disease. The Mediterranean diet showed superior efficacy in reducing recurrent MACE while improving insulin sensitivity, glycemic control, and lipoprotein metabolism, particularly among individuals with impaired glucose regulation [8].

Collectively, these randomized trials provide a substantial body of evidence supporting the Mediterranean diet in both primary and secondary cardiovascular prevention.

### 4.2. Effects on Lipid Metabolism

One of the principal mechanisms underlying Mediterranean diet-mediated cardiovascular protection involves favorable modulation of lipid metabolism. Extra-virgin olive oil, rich in monounsaturated fatty acids and phenolic compounds, reduces circulating low-density lipoprotein cholesterol (LDL-C) while preserving or increasing high-density lipoprotein cholesterol (HDL-C). Beyond quantitative lipid changes, the Mediterranean diet also improves lipoprotein function by reducing LDL-C oxidation, enhancing HDL-mediated reverse cholesterol transport, and decreasing concentrations of small dense LDL particles that exhibit greater atherogenic potential [50]. Nuts, particularly walnuts, almonds, and hazelnuts, provide unsaturated fatty acids, phytosterols, fiber, tocopherols, and polyphenols that further improve lipid profiles and endothelial function [51].

### 4.3. Anti-Inflammatory and Antioxidant Properties

The Mediterranean dietary pattern exerts potent anti-inflammatory effects through multiple complementary pathways.

Extra-virgin olive oil contains hydroxytyrosol, oleuropein, tyrosol, oleocanthal, and numerous minor phenolic compounds capable of suppressing NF-κB activation, reducing expression of pro-inflammatory cytokines, and limiting oxidative damage [52]. Experimental studies also show that oleocanthal inhibits cyclooxygenase activity, suggesting biological actions that partially resemble those of non-steroidal anti-inflammatory drugs.

Fruits, vegetables, legumes, herbs, spices, and whole grains contribute abundant vitamins, carotenoids, flavonoids, anthocyanins, and dietary fiber that collectively reinforce endogenous antioxidant defenses. Rather than functioning as isolated free radical scavengers, these compounds activate endogenous cytoprotective pathways including nuclear factor erythroid 2-related factor 2 (Nrf2), AMPK, and sirtuin signaling, thereby improving mitochondrial resilience and cellular stress responses [53].

These mechanisms translate clinically into lower circulating concentrations of inflammatory biomarkers including C-reactive protein, IL-6, TNF-α, vascular adhesion molecules, and oxidized LDL-C particles, contributing to stabilization of vulnerable atherosclerotic plaques.

### 4.4. Endothelial Function and Vascular Protection

Multiple intervention studies have demonstrated that adherence to a Mediterranean diet significantly improves endothelium-dependent vasodilation as measured by flow-mediated dilation. Increased nitric oxide bioavailability, reduced oxidative stress, and decreased endothelial activation collectively enhance vascular function [54].

Polyphenols derived from olive oil, cocoa, berries, grapes, and red wine stimulate eNOS, improve mitochondrial efficiency within endothelial cells, and reduce endothelial apoptosis. At the same time, dietary nitrates in leafy green vegetables increase nitric oxide production through the nitrate–nitrite–NO pathway, providing an additional mechanism that supports vascular health.

The Mediterranean dietary pattern also attenuates arterial stiffness and improves microvascular function; Simultaneously, polyphenols reduce mitochondrial ROS production, improve oxidative phosphorylation efficiency, and stimulate mitophagy [55].

### 4.5. Transferability Beyond the Mediterranean Basin

An important limitation of the current evidence base is that the randomized trials establishing the cardiovascular efficacy of the Mediterranean diet, Lyon, PREDIMED, and CORDIOPREV, were all conducted in Southern European populations for whom this dietary pattern was already culturally familiar [7,8,46]. Adherence in these trials therefore reflected reinforcement of existing habits rather than adoption of an unfamiliar dietary model, and the effect estimates may not transfer directly to populations where the Mediterranean diet represents a substantial departure from customary eating [56]. Paradoxically, in recent years adherence to the traditional Mediterranean diet has declined in several Mediterranean populations, while Mediterranean-like dietary patterns have gained popularity in non-Mediterranean countries, largely because of growing recognition of their health benefits [57]. This “Mediterranean diet paradox” highlights the urgent need for coordinated public health strategies to preserve and promote traditional healthy dietary patterns within the Mediterranean region itself [56].

This paradox is compounded by two divergent trends. First, adherence to the traditional Mediterranean pattern is declining within the Mediterranean region itself, particularly among younger generations, driven by urbanization, increased availability of UPFs, and the globalization of Western dietary habits. Second, the cardiovascular burden within Europe is greatest outside the Mediterranean basin: age-standardized circulatory-disease mortality varies several-fold across the European Union, with the highest rates concentrated in Central and Eastern Europe. The populations that stand to gain most from Mediterranean-type dietary interventions are, thus, in general, those with the weakest evidence base.

Available data nevertheless suggest that adoption outside the region is achievable. Structured interventions in Northern European and Australian populations have demonstrated meaningful and sustained increases in Mediterranean diet adherence scores over 6 to 12 months, together with favorable changes in intermediate cardiometabolic markers [58]. These studies were feasibility or adherence trials and were not powered for clinical endpoints; they show that dietary change is attainable, not that the cardiovascular benefit observed in Southern European trials is reproduced elsewhere.

Trials evaluating such adaptations in high-risk, non-Mediterranean populations represent a priority research gap.

## 5. Beyond the Mediterranean Diet: Emerging Dietary Patterns for Cardiovascular Prevention

Although the Mediterranean diet remains the most extensively validated nutritional model for cardiovascular prevention, attention has increasingly turned to other dietary patterns that share common biological principles while differing in food composition, cultural background, and nutritional philosophy. Rather than representing competing strategies, these dietary approaches often converge on several key characteristics, including high consumption of plant-derived foods, reduced intake of UPFs, preferential use of unsaturated fats, and limited consumption of refined carbohydrates and processed meats. As discussed in Section 2 and Section 3, each of the dietary patterns described below may influence cardiovascular risk through both adiposity-mediated and weight-independent pathways.

### 5.1. Dietary Approaches to Stop Hypertension

The DASH diet was originally developed to reduce blood pressure through dietary modification without pharmacological intervention. Since its introduction, DASH has become one of the most extensively investigated nutritional strategies for hypertension management and cardiovascular risk reduction [59].

The DASH dietary pattern emphasizes abundant consumption of fruits, vegetables, whole grains, legumes, low-fat dairy products, nuts, and lean protein sources while restricting sodium, sugar-sweetened beverages, processed foods, and saturated fats. Compared with the Mediterranean diet, DASH generally includes lower total fat intake and places greater emphasis on sodium restriction.

The original DASH trial demonstrated clinically meaningful reductions in both systolic and diastolic blood pressure within only a few weeks of dietary intervention, independent of weight loss [9]. Subsequent studies confirmed improvements in arterial stiffness, endothelial function, insulin sensitivity, and renal function, particularly among individuals with hypertension, chronic kidney disease, and metabolic syndrome [60].

Meta-analyses further indicate that high adherence to the DASH diet is associated with reduced incidence of stroke, heart failure, coronary artery disease, and cardiovascular mortality. These benefits appear to result from improved electrolyte balance, enhanced nitric oxide bioavailability, reduced oxidative stress, and attenuated vascular inflammation [61].

Recent metabolomic investigations have identified distinct serum and urinary signatures associated with adherence to the DASH diet [61]. These findings suggest that the DASH diet’s benefits extend beyond sodium restriction and may reflect the combined influence of its high potassium, magnesium, calcium, fiber, and phytochemical content. Consistent with this interpretation, the original DASH trial showed significant blood-pressure reductions while sodium intake and body weight were held constant, whereas the subsequent DASH-Sodium trial showed that sodium restriction provided an additional benefit [59].

### 5.2. Plant-Based Dietary Patterns

Growing environmental awareness, together with accumulating clinical evidence, has substantially increased interest in plant-based nutrition. Plant-based diets encompass a broad spectrum of eating patterns, from predominantly plant-centered diets to strict vegetarian and vegan regimens. Their cardiovascular effects therefore depend largely on food quality rather than simply excluding animal-derived products.

Healthy plant-based diets rich in vegetables, legumes, fruits, nuts, seeds, whole grains, and olive oil consistently demonstrate favorable effects on blood pressure, plasma lipids, insulin sensitivity, body weight, inflammatory biomarkers, and cardiovascular risk. Conversely, plant-based diets dominated by refined grains, added sugars, sweetened beverages, and highly processed meat substitutes may not provide comparable benefits, even when meeting vegetarian or vegan criteria [62].

Prospective cohort studies involving hundreds of thousands of participants have shown inverse associations between adherence to healthy plant-based dietary indices and the incidence of coronary heart disease, hypertension, and type 2 diabetes mellitus [63,64]. These associations remain significant after adjustment for BMI, smoking status, physical activity, and socioeconomic variables, suggesting that they are not fully explained by these measured factors. However, persistence after multivariable adjustment does not establish causality, as residual confounding, measurement error in dietary assessment, reverse causation, and unmeasured socioeconomic or behavioral factors cannot be excluded [65].

Nevertheless, clinicians should recognize that strict vegan diets may increase the risk of deficiencies in vitamin B12, iron, calcium, iodine, zinc, and long-chain omega-3 fatty acids unless appropriately planned. These nutritional considerations become particularly relevant among elderly individuals and patients with established cardiovascular disease.

### 5.3. The MIND Diet

The MIND diet combines elements of the Mediterranean and DASH dietary patterns while emphasizing foods associated with preserving cognitive function [10].

Although initially developed for the prevention of neurodegenerative disorders, accumulating evidence- derived predominantly from observational studies—suggests that the MIND diet has also been associated with favorable cardiovascular and neurovascular outcomes, although the evidence is predominantly observational [66]. Green leafy vegetables, berries, whole grains, legumes, olive oil, nuts, and fish constitute its principal components, whereas processed foods, butter, pastries, red meat, and fried foods are strongly discouraged.

Given the close relationship between vascular disease and cognitive impairment, it is plausible that many mechanisms protecting cerebral function simultaneously reduce cardiovascular risk. Improved endothelial function, reduced systemic inflammation, and enhanced mitochondrial activity indicate that high adherence to the MIND diet is associated with lower incidence of stroke, atrial fibrillation, heart failure, and vascular dementia. However, randomized controlled trials specifically evaluating cardiovascular outcomes remain relatively limited compared with the evidence available [66].

### 5.4. Nordic Diet

The Nordic dietary pattern is a regionally adapted dietary model that shares many plant-based and sustainability-oriented characteristics with the Mediterranean diet while emphasizing foods traditionally available and consumed in Northern Europe [67].

Typical components include whole grains (particularly rye, barley, and oats), berries, root vegetables, legumes, rapeseed oil, fatty fish and fermented dairy products. Similarly to the Mediterranean diet, the Nordic dietary pattern emphasizes minimally processed foods rich in fiber, omega-3 fatty acids, antioxidants, and phytochemicals, thereby promoting multiple cardiometabolic benefits simultaneously of processed meat and refined carbohydrates [68].

Although evidence regarding hard cardiovascular endpoints remains less extensive than for Mediterranean dietary interventions, current findings support the Nordic diet as a culturally appropriate dietary model particularly for Nordic and Scandinavian populations, reflecting regional food availability and traditional dietary habits [11,67,69].

### 5.5. Western Dietary Pattern

In contrast to the dietary patterns described above, the Western dietary pattern is typically characterized by high consumption of refined carbohydrates, processed and red meats, sugar-sweetened beverages, foods rich in saturated fat and sodium, and a high proportion of UPFs, together with low intake of fruits, vegetables, legumes, and whole grains.

Large prospective cohort studies consistently associate high UPFs consumption with obesity, hypertension, type 2 diabetes mellitus, coronary artery disease, stroke, heart failure, and increased all-cause mortality [70]. These associations often persist after adjustment for total caloric intake; however, this does not establish an independent causal effect of food processing, because residual confounding and dietary-measurement error remain.

Several mechanisms have been proposed. UPFs generally exhibit high energy density, low satiety, rapid glycemic responses, poor micronutrient content, altered food matrices, and reduced dietary fiber. Additionally, certain additives may impair gut barrier integrity, modify microbial composition, and enhance systemic inflammatory responses [18]. However, the UPFs category is heterogeneous and may include products with substantially different nutritional compositions, including some plant-based or specialty foods. Therefore, food-processing classification should not be interpreted as a direct surrogate for nutritional quality, and the cardiovascular implications of individual UPFs may differ by nutrient composition, food matrix, and consumption patterns.

More broadly, accumulating evidence across nutritional epidemiology and intervention studies has shifted nutritional science away from reductionist approaches focused exclusively on individual nutrients toward a more holistic evaluation of dietary patterns, food quality, and degree of food processing. These dimensions should be considered complementary, but conceptually distinct, components of dietary assessment. Table 2 summarizes the main evidence supporting dietary patterns beyond the Mediterranean diet, including predominant study designs, principal cardiovascular findings, and major limitations.

## 6. Nutrition in Established Cardiovascular Disease: Secondary Prevention and Obesity Management

### 6.1. Nutritional Management

The role of nutrition changes once cardiovascular disease is established. Whereas dietary interventions in primary prevention principally aim to prevent cardiovascular events through long-term modification of cardiometabolic risk, nutritional therapy in patients with established CVD forms part of a broader secondary-prevention and disease-management strategy. In this setting, treatment goals extend beyond prevention of weight gain or achievement of weight loss.

Obesity is particularly relevant in this population because excess and dysfunctional adiposity may amplify several mechanisms driving recurrent cardiovascular events. Nutritional management should therefore address both energy balance and dietary quality and integrate physical activity, behavioral interventions, evidence-based pharmacotherapy, and conventional cardiovascular risk-factor management (Figure 3).

Evidence supporting nutritional therapy in patients with established CVD comes primarily from secondary-prevention trials. The Lyon Diet Heart Study demonstrated a marked reduction in recurrent cardiovascular events after myocardial infarction with a Mediterranean-type dietary intervention, while CORDIOPREV showed that a Mediterranean diet reduced recurrent MACE compared with a low-fat diet in patients with established coronary heart disease. Importantly, these findings indicate that nutritional management in established CVD should not be viewed solely as a strategy for weight reduction, but as a component of comprehensive secondary prevention capable of modifying residual cardiometabolic risk. Combining nutritional therapy with modern anti-obesity pharmacotherapy represents an emerging area of cardiovascular prevention. Recent trials of glucagon-like peptide-1 receptor agonists have shown substantial weight reduction and improved cardiovascular outcomes, suggesting that future obesity treatment will likely involve integrated approaches combining pharmacological, nutritional, and lifestyle interventions [24].

Broader evidence syntheses support these findings. A network meta-analysis of 17 randomized trials including 6331 patients with established CVD found potentially clinically relevant effects of dietary interventions on body weight and blood pressure, although substantial heterogeneity and attenuation of effects over time precluded identifying a single optimal dietary pattern [74]. More recent systematic review and meta-analysis evidence also supports Mediterranean dietary adherence in secondary prevention, with reductions in recurrent cardiovascular events and mortality among patients with established CVD [73]. Sustained adherence is necessary to maintain dietary exposure, although direct evidence linking long-term adherence itself to hard cardiovascular outcomes remains limited [58,75].

### 6.2. From Weight Loss to Metabolic Health

Individuals with obesity exhibit considerable heterogeneity: some develop severe metabolic complications, whereas others maintain relatively preserved insulin sensitivity and cardiovascular profiles despite increased adiposity. This concept, often referred to as *metabolically healthy obesity*, remains controversial but highlights the importance of evaluating the metabolic phenotype rather than BMI alone [76]. However, the clinical–preclinical distinction offers a more operational framework for the same underlying observation [3] (Table 3).

A patient-centered approach should therefore move beyond the traditional focus on kilograms lost and instead emphasize restoration of metabolic health. In this context, evidence-based nutritional strategies should address both energy balance and diet quality, including increased consumption of minimally processed plant-derived foods and dietary fiber, preferential use of unsaturated fats, limitation of ultra-processed foods, refined carbohydrates, excess sodium, and saturated fats, and adequate protein intake to preserve lean body mass. These interventions should be individualized according to cardiometabolic status, comorbidities, nutritional requirements, and treatment goals.

Successful implementation of these nutritional strategies requires multidisciplinary collaboration among cardiologists, endocrinologists, registered dietitians and nutrition specialists, primary care physicians, psychologists, and exercise professionals [77]. Taken together, the available evidence supports a distinction between weight loss and broader improvement in cardiometabolic health. Cardiovascular benefit may occur through reductions in visceral and ectopic adiposity and improvements in blood pressure, lipid profile, glycemic control, inflammation, endothelial function, and dietary quality, even when changes in total body weight are modest. Conversely, reductions in body weight do not necessarily capture the full biological impact of nutritional interventions. These observations support a multidimensional approach to obesity management in cardiovascular disease, in which body weight remains clinically relevant but is interpreted alongside adiposity distribution, metabolic risk factors, functional status, and diet quality.

### 6.3. Imaging Biomarkers of Adipose Tissue Distribution

Waist circumference does not distinguish subcutaneous from visceral compartments, nor does it capture fat depots in direct anatomical contact with the myocardium. Epicardial adipose tissue (EAT), located between the myocardium and the visceral pericardium, has attracted attention for its continuity with the coronary arteries and its local paracrine activity [78]. Echocardiography can quantify it by thickness, while computed tomography and cardiac magnetic resonance can quantify it volumetrically. In a systematic review and meta-analysis of 29 studies comprising 19,709 patients, increased EAT thickness and volume were independent predictors of major adverse cardiovascular events across imaging modalities [79]. Automated deep-learning quantification from coronary computed tomography angiography has more recently linked EAT volume and attenuation to subsequent myocardial infarction [80]. The results of this study are still preliminary, given the lack of standardized cut-offs, significant inter-study heterogeneity, and no prospective trial validating EAT-based risk stratification. EAT is therefore most appropriately regarded as a biologically informative marker rather than a clinical tool. Its potential as an intermediate endpoint indicates its relevance to nutritional cardiology, as ectopic fat depots respond to dietary interventions more readily than total body weight. This pattern is consistent with the dissociation between weight loss and cardiovascular benefit observed in Mediterranean diet trials.

### 6.4. Integrating Incretin-Based Pharmacotherapy with Nutritional Therapy

Glucagon-like peptide-1 receptor agonists (GLP-1 RAs) and dual glucose-dependent insulinotropic polypeptide (GIP)/GLP-1 receptor agonists now achieve weight reduction previously addressed only through bariatric surgery. Semaglutide reduced MACE in patients with overweight or obesity and established CVD without diabetes, with benefit only partially explained by the extent of weight loss [24], while tirzepatide proved non-inferior to dulaglutide in type 2 diabetes mellitus, with a significant reduction in an expanded composite endpoint [23]. No placebo-controlled cardiovascular outcome trial of a dual agonist in obesity without diabetes has yet been reported, and SURMOUNT-MMO is designed to address this gap [81].

Effective pharmacotherapy does not diminish nutritional therapy but redefines its objective. Body-composition substudies consistently show that a clinically relevant proportion of weight lost during incretin-based treatment derives from fat-free mass, with estimates of 30–40%, a concern particularly in older patients and in sarcopenic obesity [82]. Reduced energy intake, early satiety, and gastrointestinal intolerance further predispose to inadequate protein, fiber, and micronutrient intake [83]. Current guidance accordingly recommends protein intake of approximately 1.2–1.6 g/kg/day in adults without chronic kidney disease, distributed across meals, combined with resistance training. However, these targets are extrapolated from bariatric surgery protocols, and prospective trials defining the optimal protein dose during incretin therapy are lacking [82].

## 7. Future Perspectives: Toward Precision Cardiovascular Nutrition

Despite substantial advances in nutritional epidemiology, marked interindividual variability persists in metabolic responses to identical dietary interventions. Precision nutrition seeks to account for this heterogeneity by integrating genetic, metabolomic, microbial, and phenotypic data into individualized dietary recommendations (Figure 4).

A major unresolved question is therefore whether individualized dietary strategies can improve cardiovascular outcomes beyond the benefits achievable through established healthy dietary patterns and conventional weight-management approaches.

Each of these data layers exists at a varying level of development. Nutrigenetic studies have uncovered various gene–diet interactions that affect lipid metabolism, body fat, and cardiometabolic risk [42]. However, the primary challenge for clinical implementation is insufficient replication of these interactions across diverse populations, as noted in an American Heart Association statement: despite significant interest, evidence for the clinical use of nutrigenomics remains inadequate. Metabolomic profiling has showed more efficiency in delivering insights, offering objective indicators of dietary compliance and revealing metabolite patterns linked to blood pressure responses, as evidenced in the DASH and DASH-Sodium studies [84]. Microbiome-centric methodologies have shown that tailored predictive models integrating microbial makeup, clinical factors, and lifestyle attributes can effectively forecast individual glycemic reactions to particular foods [85]. However, the gut microbiota remains a potential, rather than confirmed, therapeutic target, as intervention trials with clinical cardiovascular outcomes remain limited.

Digital technologies are expected to accelerate this field. Continuous glucose monitoring, wearable biosensors, automated dietary assessment, and machine-learning models applied to imaging and multiomic data may enable dynamic adaptation of dietary recommendations based on biological feedback rather than fixed, generalized guidelines [86]. These tools also address a persistent practical limitation of nutritional intervention, namely the difficulty of monitoring adherence and sustaining behavioral change over time.

## 8. Conclusions

Obesity and CVD are closely interconnected conditions shaped by adipose tissue dysfunction, chronic low-grade inflammation, oxidative stress, mitochondrial impairment, and gut microbiota alterations. Diet influences cardiovascular risk not only through energy balance, but also through direct modulation of these pathways, a distinction with practical consequences, since dietary quality warrants clinical attention independently of the degree of weight loss achieved.

The Mediterranean diet has one of the most developed randomized evidence bases among dietary patterns for cardiovascular prevention, including trials with adjudicated cardiovascular endpoints in both primary and secondary prevention. However, the strength of evidence varies across dietary patterns and according to whether the outcomes considered are clinical cardiovascular events or intermediate cardiometabolic markers. These trials were conducted predominantly in Southern European populations, and their transferability elsewhere requires further study. The DASH, healthy plant-based, Nordic, and MIND patterns share the same core principles and consistently improve intermediate cardiometabolic markers, although evidence for hard endpoints rests largely on observational data. Conversely, less healthy dietary patterns may encompass several distinct exposures, including high consumption of UPFs, refined carbohydrates, saturated fats, and sodium. These factors should not be considered interchangeable, as they represent different dimensions of diet and are supported by different combinations of observational, interventional, and mechanistic evidence.

Clinically, these observations argue for assessment beyond BMI and treatment goals that encompass metabolic health alongside weight loss, using the multidimensional cardiometabolic parameters described above, rather than body weight in isolation. Precision nutrition offers a plausible route to addressing interindividual variability in dietary response, but has not yet been tested against clinical cardiovascular endpoints. Meanwhile, the core principles remain consistent: minimally processed plant-derived foods, high-quality unsaturated fats, adequate fiber, limited UPF consumption, and dietary patterns that can be sustained over time. Diet should therefore be regarded as a modifiable determinant of cardiovascular risk that acts through definable biological mechanisms and deserves the same clinical attention as other established risk factors.

## Figures and Tables

**Figure 1:**
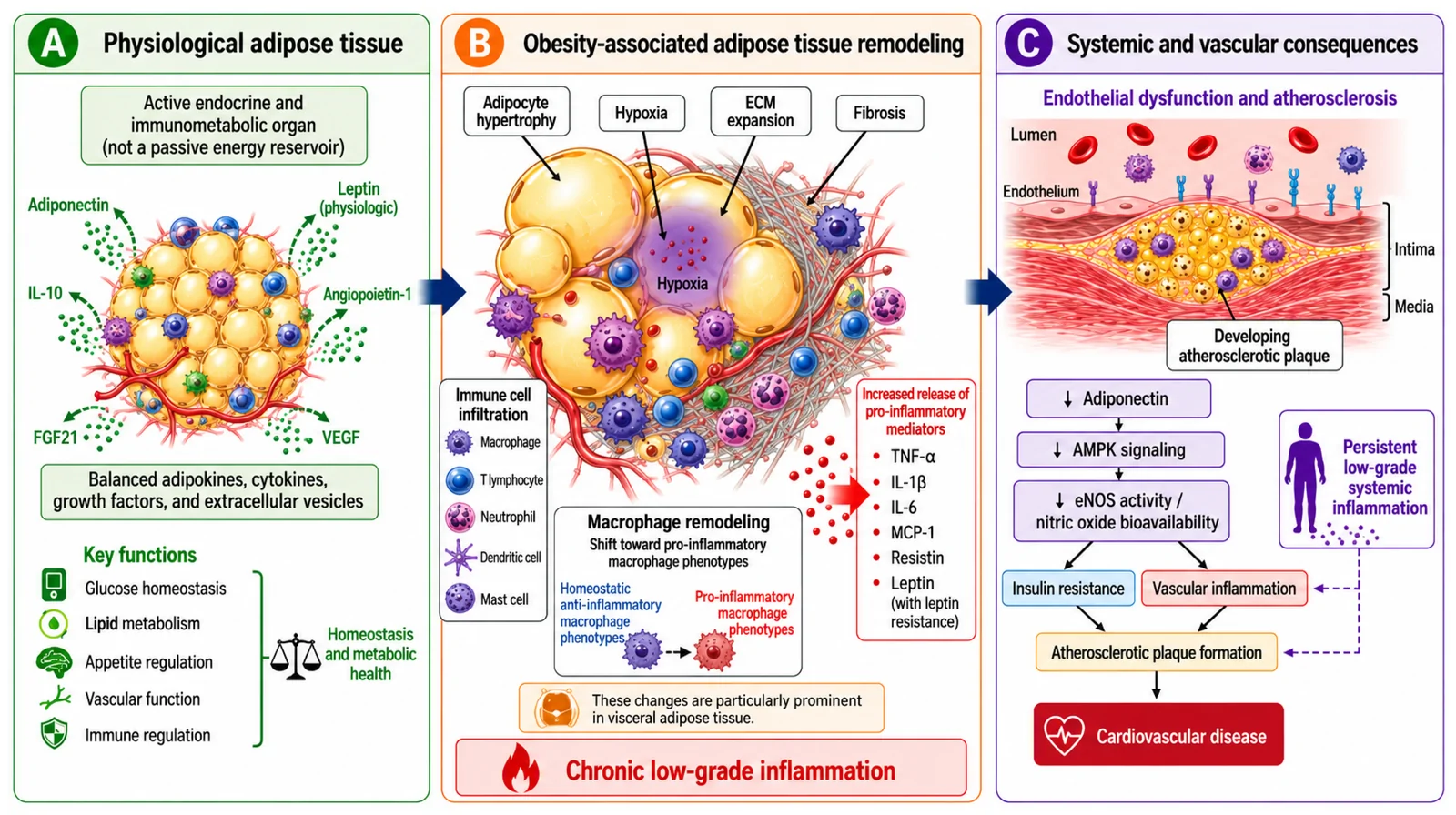
**Adipose tissue dysfunction: a link between obesity and cardiovascular disease.** (**A**). Under physiological conditions, adipose tissue acts as an active endocrine and immunometabolic organ, contributing to glucose and lipid homeostasis (**B**). During obesity, adipocyte hypertrophy, local hypoxia, extracellular matrix expansion, fibrosis, and immune-cell infiltration promote a shift toward pro-inflammatory macrophage phenotypes and increase the release of inflammatory mediators. (**C**). At the same time, reduced adiponectin levels impair AMPK signaling and endothelial nitric oxide synthase activity, decrease nitric oxide bioavailability, and promote insulin resistance, vascular inflammation, endothelial dysfunction, atherosclerotic plaque formation, and, ultimately, cardiovascular disease. **Abbreviations:** AMPK, AMP-activated protein kinase; ECM, extracellular matrix; eNOS, endothelial nitric oxide synthase; FGF21, fibroblast growth factor 21; IL, interleukin; MCP-1, monocyte chemoattractant protein-1; TNF-α, tumor necrosis factor alpha; VEGF, vascular endothelial growth factor.

**Figure 2:**
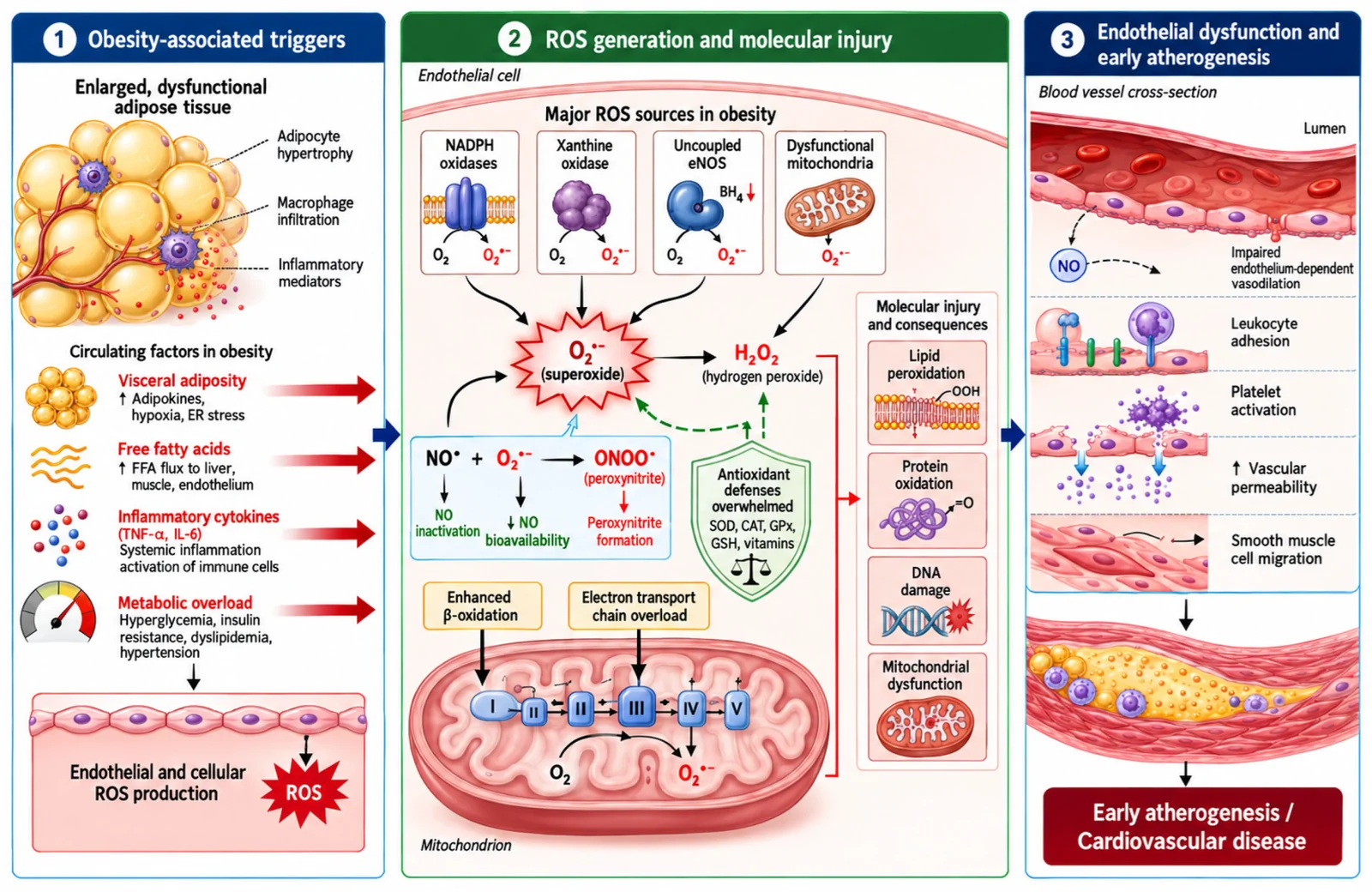
**Oxidative stress as a molecular link between obesity and cardiovascular disease.** Visceral adiposity, elevated free fatty acids, chronic inflammation, and metabolic overload increase reactive oxygen species generation. When antioxidant defenses are exceeded, oxidative damage affects lipids, proteins, DNA, and mitochondrial function. In endothelial cells, superoxide reduces NO bioavailability and promotes peroxynitrite formation, impairing vasodilation and inducing leukocyte adhesion, platelet activation, increased vascular permeability, and smooth muscle cell migration. **Abbreviations:** BH_4_, tetrahydrobiopterin; CAT, catalase; CVD, cardiovascular disease; DNA, deoxyribonucleic acid; eNOS, endothelial nitric oxide synthase; ER, endoplasmic reticulum; FFA, free fatty acids; GPx, glutathione peroxidase; GSH, reduced glutathione; H_2_O_2_, hydrogen peroxide; IL-6, interleukin-6; NADPH, nicotinamide adenine dinucleotide phosphate; NO, nitric oxide; O_2_, molecular oxygen; O_2_•−, superoxide anion; ONOO−, peroxynitrite; ROS, reactive oxygen species; SOD, superoxide dismutase; TNF-α, tumor necrosis factor alpha.

**Figure 3:**
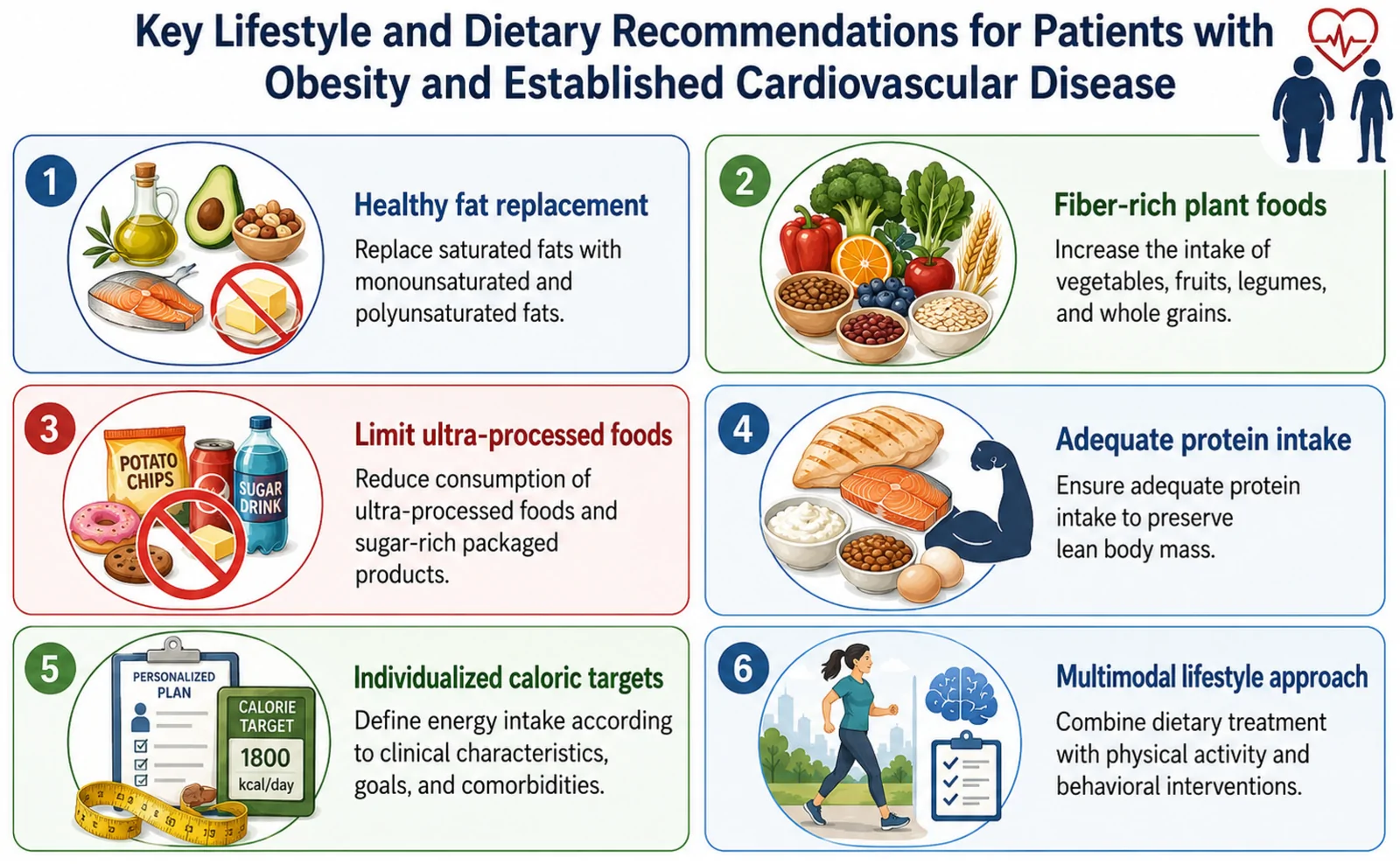
**Dietary and lifestyle recommendations for patients with obesity and established cardiovascular disease.** Recommended management should prioritize replacing saturated fats with monounsaturated and polyunsaturated fats, increasing intake of fiber-rich plant foods, reducing ultra-processed foods, ensuring adequate protein intake to preserve lean body mass, and setting individualized caloric targets. These dietary measures should be integrated with physical activity and behavioral interventions within a comprehensive cardioprotective strategy [8,73,74].

**Figure 4:**
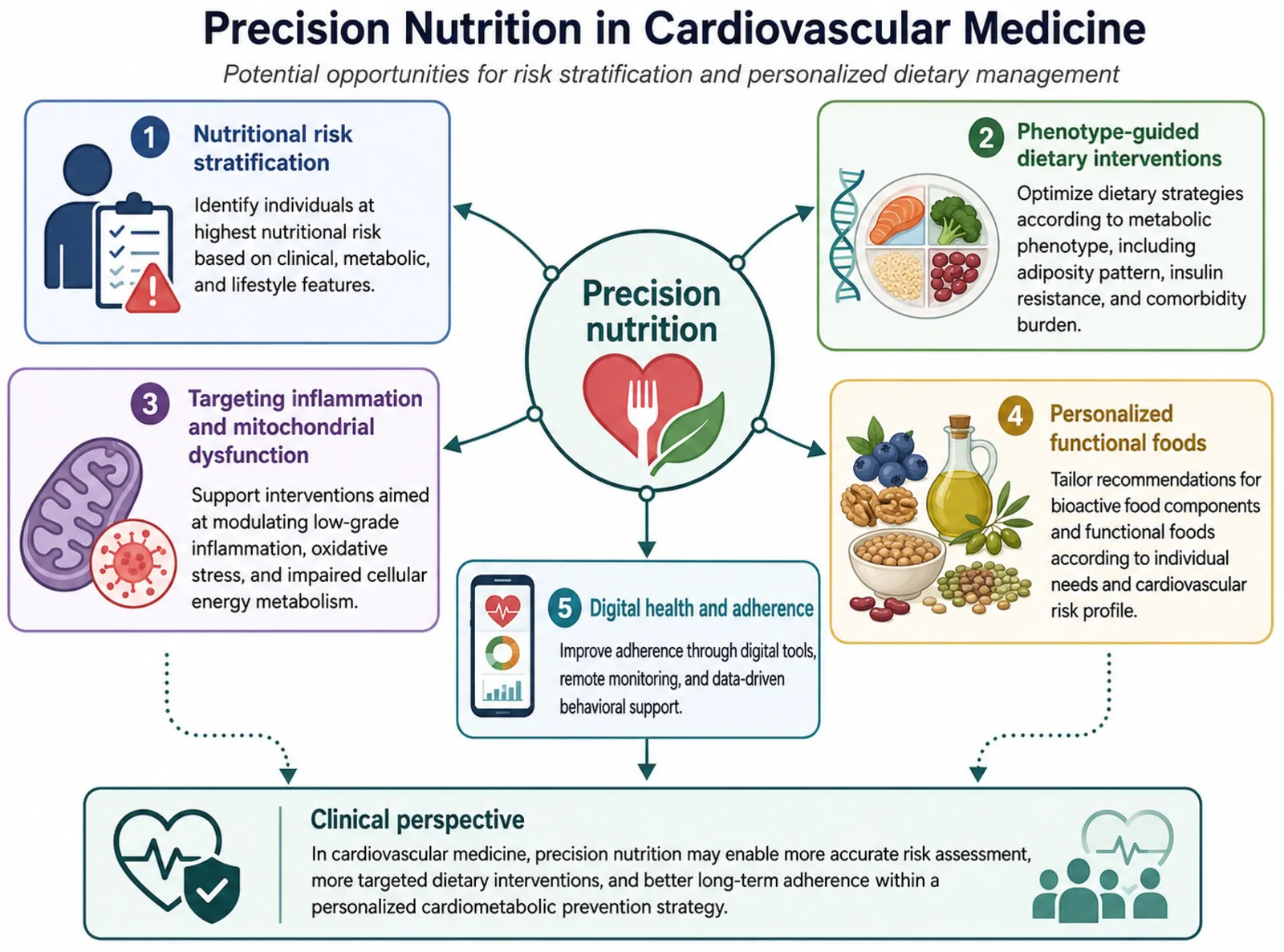
**Mechanistic framework of precision nutrition in cardiovascular medicine.** Precision nutrition integrates clinical, metabolic, phenotypic, and lifestyle data to identify individuals at increased nutritional and cardiovascular risk and to guide tailored dietary interventions. By accounting for adiposity distribution, insulin resistance, comorbidity burden, inflammatory status, oxidative stress, and mitochondrial dysfunction, this approach may help select targeted nutrients, bioactive compounds, and functional foods. Digital health technologies may further enhance monitoring, behavioral support, and long-term adherence, thereby promoting more individualized cardiometabolic prevention and management.

**Table 1:** Major randomized trials investigating the cardiovascular effects of Mediterranean dietary patterns in primary and secondary cardiovascular prevention.

Study	Population and Design	Dietary Intervention	Comparator	Follow-Up	Main Cardiovascular Outcomes	Principal Findings
**Lyon Diet Heart Study**	605 patients with a previous myocardial infarction; randomized secondary-prevention trial	Mediterranean-type diet enriched in α-linolenic acid	Prudent Western-type diet	Mean 46 months	Cardiac death, non-fatal myocardial infarction, unstable angina, stroke, heart failure and other cardiovascular events	Cardiac death or non-fatal myocardial infarction occurred in 14 patients in the Mediterranean-diet group versus 44 controls. Broader cardiovascular composite endpoints were also markedly reduced, supporting a substantial benefit in secondary prevention [46].
**PREDIMED pilot study**	772 adults at high cardiovascular risk; multicenter randomized trial	Mediterranean diet supplemented with either extra-virgin olive oil or mixed nuts	Advice to follow a low-fat diet	3 months	Cardiovascular risk factors	Both Mediterranean-diet interventions improved blood pressure, glucose regulation, lipid profiles and selected inflammatory markers compared with the low-fat control diet; the study was not designed to assess clinical cardiovascular events [47].
**PREDIMED**	7447 adults at high cardiovascular risk without established cardiovascular disease; multicenter randomized primary-prevention trial	Energy-unrestricted Mediterranean diet supplemented with extra-virgin olive oil or mixed nuts	Advice to reduce dietary fat	Median approximately 4.8 years	Composite of cardiovascular death, myocardial infarction and stroke	Compared to the control diet, the adjusted HR for major cardiovascular events was 0.69 (95% CI 0.53–0.91) with extra-virgin olive oil and 0.72 (95% CI 0.54–0.95) with nuts, corresponding to an approximately 28–31% relative risk reduction [7].
**CORDIOPREV—atherosclerosis substudy**	939 patients with established coronary heart disease from the CORDIOPREV trial	Mediterranean diet rich in extra-virgin olive oil	Low-fat diet rich in complex carbohydrates	5–7 years	Carotid intima–media thickness and carotid plaque burden	The Mediterranean diet reduced carotid intima–media thickness at 5 years, with the effect maintained at 7 years, and produced greater reductions in carotid plaque height than the low-fat diet, indicating slower atherosclerotic progression [48].
**CORDIOPREV**	1002 patients with established coronary heart disease; single-center randomized secondary-prevention trial	Mediterranean diet	Low-fat diet	7 years	Composite of myocardial infarction, revascularization, ischemic stroke, peripheral artery disease and cardiovascular death	The primary endpoint occurred in 87 participants assigned to the Mediterranean diet and 111 assigned to the low-fat diet. Adjusted HRs ranged from 0.719 to 0.753, indicating an approximately 25–28% lower risk of major cardiovascular events [8].
**PREDIMED-Plus**	6874 older adults with overweight or obesity and metabolic syndrome; multicenter randomized lifestyle trial	Energy-reduced Mediterranean diet combined with physical activity and behavioral support	Ad libitum Mediterranean diet without specific weight-loss or physical-activity intervention	Interim analyses at 1–3 years; long-term cardiovascular follow-up ongoing	Adiposity, body composition and cardiovascular risk factors	The intensive intervention produced greater reductions in body weight, total fat and visceral adiposity and attenuated loss of lean mass. These findings support cardiometabolic benefit, although published interim analyses were not designed to establish an independent effect of the diet on hard cardiovascular events [49].

CI, confidence interval; HR, hazard ratio; MI, myocardial infarction.

**Table 2:** Summary of the evidence supporting dietary patterns beyond the Mediterranean diet.

Dietary Pattern	Main Evidence Base	Cardiovascular Findings	Main Limitations
**DASH** [59]	RCTs for blood pressure and cardiometabolic risk factors; observational studies/meta-analyses for clinical CVD outcomes	Consistent reduction in blood pressure; favorable effects on vascular and metabolic risk factors; higher adherence associated with lower CVD, stroke and heart failure risk	Limited randomized evidence for hard cardiovascular endpoints
**Healthy plant-based diets** [71]	Predominantly prospective cohort studies and meta-analyses	Higher adherence associated with lower CVD risk; associations appear stronger for nutritionally healthy plant-based patterns	Observational evidence; heterogeneous definitions; residual confounding and dietary-measurement error
**Vegetarian/vegan diets** [72]	Prospective cohort studies and meta-analyses	Lower overall CVD and ischemic heart disease risk; less consistent evidence for stroke	Limited randomized cardiovascular outcome evidence; substantial heterogeneity between dietary definitions
**MIND diet** [66]	Predominantly observational studies	Associations reported with several cardiovascular and neurovascular outcomes	Limited cardiovascular-specific RCT evidence; originally developed for cognitive rather than cardiovascular outcomes
**Nordic diet** [11,69]	Prospective cohorts for clinical outcomes; RCTs for intermediate risk factors	Lower CVD incidence/mortality in observational analyses; improvements in LDL-C, blood pressure, glucose/insulin and body weight in RCTs	Hard cardiovascular endpoints remain predominantly observational; relatively few large long-term intervention studies
**Western diet/high UPF consumption** [70]	Large prospective cohorts and meta-analyses	Higher intake associated with obesity, diabetes, CVD, stroke, heart failure and mortality	Predominantly observational evidence; heterogeneity of UPF classification and potential residual confounding

**Table 3:** Key parameters for comprehensive cardiometabolic assessment in patients with obesity and cardiovascular disease.

Assessment Domain	Clinical Relevance
Waist circumference	Provides a simple surrogate measure of central adiposity and cardiometabolic risk.
Visceral adiposity	Reflects metabolically active fat accumulation associated with insulin resistance, inflammation, and cardiovascular risk.
Glycemic control	Identifies impaired glucose metabolism and supports assessment of diabetes-related cardiovascular risk.
Lipid profile	Evaluates atherogenic lipid abnormalities and guides lipid-lowering strategies.
Inflammatory biomarkers	May help characterize the chronic low-grade inflammatory state associated with obesity.
Physical fitness	Provides an integrated measure of functional capacity and is independently associated with cardiovascular prognosis.
Epicardial adipose tissue	Metabolically active depot in direct contact with the myocardium and coronary arteries; associated with major adverse cardiovascular events, although standardized thresholds are lacking
Diet quality and dietary pattern	Assesses adherence to cardioprotective dietary patterns, intake of minimally processed plant-derived foods, dietary fiber and unsaturated fats, and consumption of ultra-processed foods, saturated fats, refined carbohydrates, and sodium, thereby helping to guide individualized nutritional interventions.

## Data Availability

No new data were created or analyzed in this study. Data sharing does not apply to this article.

## Abbreviations

The following abbreviations are used in this manuscript:

AMPK: AMP-activated protein kinase; BMI: body mass index; CI: confidence interval; CVD: Cardiovascular disease; DALY: disability-adjusted life-year; DAMP: danger-associated molecular patterns; DASH: Dietary Approaches to Stop Hypertension; DNA: deoxyribonucleic acid; EAT: epicardial adipose tissue; eNOS: endothelial nitric oxide synthase; GIP: glucose-dependent insulinotropic polypeptide; GLP-1 RA: glucagon-like peptide-1 receptor agonist; HDL-C: high-density lipoprotein cholesterol; HR: hazard ratio; IL-1β: interleukin-1β; IL-6: interleukin-6; LDL-C: low-density lipoprotein cholesterol; MACE: major adverse cardiovascular events; MCP-1: monocyte chemoattractant protein-1; MIND: Mediterranean-DASH Intervention for Neurodegenerative Delay; NADPH: nicotinamide adenine dinucleotide phosphate; NF-kB: nuclear factor-kappa B; NLRP3: nucleotide-binding oligomerization domain-like receptor protein 3; NO: nitric oxide; PGC-1α: peroxisome proliferator-activated receptor gamma coactivator-1α; ROS: reactive oxygen species; SCFAs: short-chain fatty acids; SIRT1: sirtuin-1; TLR4: Toll-like receptor 4; TMAO: trimethylamine-N-oxide; TNF-α: tumor necrosis factor-α; UPFs: ultra-processed foods.
